# Cost-effectiveness of Direct Oral Anticoagulant vs. Warfarin Among Atrial Fibrillation Patients With Intermediate Stroke Risk

**DOI:** 10.3389/fcvm.2022.849474

**Published:** 2022-04-11

**Authors:** Ju Hee Choi, Woojin Kim, Yun Tae Kim, Jaelim Cho, Seung Yong Shin, Changsoo Kim, Jin-Bae Kim

**Affiliations:** ^1^Department of Public Health, Graduate School, Yonsei University, Seoul, South Korea; ^2^Department of Preventive Medicine, Yonsei University College of Medicine, Seoul, South Korea; ^3^Division of Cardiology, Chung-Ang University Hospital, Seoul, South Korea; ^4^Institute of Human Complexity and Systems Science, Yonsei University, Incheon, South Korea; ^5^Division of Cardiology, Department of Internal Medicine, Kyung Hee University Hospital, Kyung Hee University, Seoul, South Korea

**Keywords:** atrial fibrillation, cost-effectiveness, anticoagulants, warfarin, intermediate stroke risk

## Abstract

**Background:**

Several studies have shown the cost-effectiveness of direct oral anticoagulants (DOACs), compared with warfarin, to prevent atrial fibrillation (AF) related complications. However, few have reported cost-effectiveness of DOACs in AF patients with intermediate stroke risk. Thus, we investigated the cost-effectiveness of DOACs vs. warfarin in non-valvular AF patients with intermediate stroke risk using national representative data.

**Methods:**

We identified 7,954 newly diagnosed non-valvular AF patients (≥18 years) with intermediate stroke risk (CHA_2_DS_2_-VASc score: 1 for men and 2 for women) using the national healthcare utilization data from August 1, 2016, to July 31, 2019. Annual incidence rate of AF-related composite outcomes (heat failure, myocardial infarction, ischemic stroke, intracerebral hemorrhage, and gastrointestinal bleeding) was estimated. Cost-effectiveness was estimated using a Markov chain model with the transition probability of 1 year. The willingness-to-pay (WTP) was set at $32,000 per quality-adjusted life-year (QALY) gained.

**Results:**

The total cost of warfarin, rivaroxaban, apixaban, dabigatran and edoxaban was $2,874, $5,761, $5,151, $5,761 and $5,851, respectively. The QALYs gained were 10.83, 10.95, 11.10, 10.49 and 10.99 years, respectively. The incremental cost-effectiveness ratio of rivaroxaban, apixaban, dabigatran and edoxaban was $29,743.99, $8,426.71, -$8,483.04 and $18,483.55, respectively. The WTP was set at $32,000. DOACs (except dabigatran) were more cost-effective compared with warfarin because they did not exceed the WTP in the base-case analysis.

**Conclusion:**

Our findings showed that DOACs were more cost-effective than warfarin in non-valvular AF patients with intermediate stroke risk.

## Introduction

The global incidence and prevalence of atrial fibrillation (AF) have increased rapidly in elderly people (1). In the Republic of Korea, AF prevalence is estimated to be 2.1% among those aged more than 65 years, and it is expected to rise to 5.8% by 2060 due to rapid population aging (2). Accordingly, the risk of complications (including stroke) and the related cost of treating AF have increased steadily (3). In the United States, the estimated incremental cost for treating AF patients reached $26 billion during 2004–2006 (4). In Europe, AF-related costs increased up to €3,000 per patient-year from 1990 to 2009 (5).

Use of anticoagulants is of importance in preventing cardiovascular complications in patients with AF. AF increases the risk of developing a thrombus due to turbulent flow in the left atrium. The thrombus from the left atrium can cause embolization in a primary organ or tissue, leading to complications such as stroke or systemic embolism, myocardial infarction (MI), and heart failure (HF) (6–8). To prevent these complications, vitamin K antagonists or direct oral anticoagulants (DOACs; rivaroxaban, apixaban, dabigatran, and edoxaban) are prescribed to patients with AF.

Based on mounting evidence on the DOACs had more cost-effective than warfarin (9–12), guidelines in Europe and the USA recommend preferential use of DOACs over warfarin to prevent cardiovascular complications in patients with AF (13, 14). In line with this, use of DOACs to prevent stroke is subsidized in AF patients since 2015 in the Republic of Korea (15). However, this reimbursement scheme is limited to AF patients with high stroke risk (defined as a CHA_2_DS_2_-VASc score of 2 or higher), as most previous studies on the cost-effectiveness of DOACs included AF patients with high stroke risk or did not consider the risk of stroke (16). Some studies have investigated the cost-effectiveness of DOACs after a stratification on individuals regarding stroke risk (9, 17, 18). To the best of our knowledge, no purposely designed study has investigated the cost-effectiveness of DOACs by focusing on the intermediate stroke risk group.

Therefore, this study investigated the cost-effectiveness of DOACs and warfarin among AF patients with intermediate stroke risk by using national representative data.

## Materials and Methods

### Data Source

In this study, we used data from the Health Insurance Review and Assessment (HIRA) on the demographics (age and sex), diagnosis [International Classification of Disease 10th Revision (ICD-10) codes and date of diagnosis], in-hospital mortality, information on prescriptions (date and chemical name), and cost. Because the HIRA database covers almost 98% of the total population (15), sampling or selection bias was minimized.

This study was approved by the Institutional Review Board of Yonsei University Health System (2021–1748–001), and informed consent was waived.

### Study Population

Of the 781,583 individuals who visited hospitals with the AF code (ICD-10: I48) between August 1, 2016, and July 31, 2019, we further identified 7,954 non-valvular AF patients using the following inclusion criteria (Supplementary Figure 1):

Individuals who were not diagnosed with AF (or valvular AF) and AF-related complications from August 1, 2015, to July 31, 2016;
Individuals who visited outpatient clinics at least twice or were admitted to a hospital at least once for AF (ICD-10: I48, I48.0, and I48.1);
Adults (aged ≥ 18 years) who took warfarin or DOACs (rivaroxaban, apixaban, dabigatran and edoxaban) and we excluded AF patients with prescriptions of both DOACs and warfarin during the study period;
Individuals with intermediate stroke risk (defined as the CHA2DS2-VASc score of 1 in men or 2 in women).

### CHA_2_DS_2_-VASc Score

To calculate the CHA_2_DS_2_-VAS_c_ score (an indicator of stroke risk), this study set the index date for each participant. The index date is defined as the date of admission or of the second outpatient visit for AF, whichever came earlier. The CHA_2_DS_2_-VAS_c_ score was calculated based on the demographics on the index date and healthcare utilization 1 year prior to the index date. The CHA_2_DS_2_-VAS_c_ score is the sum of the scores of each component. One point was given to those who aged 65–75 years; those with a history of hypertension, diabetes, congestive HF, or vascular disease (MI or a peripheral vascular disease); and women. Two points were given to those aged 75 years or older and those with a history of stroke or systemic embolism. The ICD-10 codes used for calculating the CHA_2_DS_2_-VAS_c_ score are presented in Table 1.

**Table 1:** Definition of CHA_2_DS_2_-VAS_c_ score components (ICD-10).

**Disease**	**ICD-10 code**	
Hypertension	I10, I11, I12, I13, I15	Essential (primary) hypertension, hypertensive heart disease, hypertensive renal disease, hypertensive heart and renal disease, secondary hypertension
Heart failure	I50	Heart failure
Stroke (IS, ICH)	I60, I61, I62, I63, I64	Subarachnoid hemorrhage, intracerebral hemorrhage, other non-traumatic intracranial hemorrhage, cerebral infarction, stroke, not specified as hemorrhage or infarction
Myocardial infarction	I21, I22	Acute myocardial infarction, subsequent myocardial infarction
Peripheral artery disease	I70, I71	Atherosclerosis, aortic aneurysm and dissection

*IS, ischemic stroke; ICH, intracranial hemorrhage*.

### Composite Outcome Measure

In this study, we calculated annual rates of the composite outcome of the following AF-related complications: HF, MI, ischemic stroke (IS), intracerebral hemorrhage (ICH), and gastrointestinal (GI) bleeding. For each composite outcome, there were records of outpatient visits at least twice or admission at least once. If the definition is satisfied no matter how many times an event occurs within a year, the analysis was conducted with one event occurrence. The ICD-10 codes used for calculating the composite outcome are presented in Table 2.

**Table 2:** Definition of health event (ICD-10).

**Disease**	**ICD-10 code**		**Inclusion criteria**
Heart failure	I50	Heart failure	Admission ≥1 or outpatient department ≥2
Myocardial infarction	I21	Acute myocardial infarction	Admission ≥1 or outpatient department ≥2
	I22	Subsequent myocardial infarction	
Ischemic stroke	I63	Cerebral infarction	Admission ≥1 or outpatient department ≥2 + brain imaging (CT, MRI)
Intracranial hemorrhage	I60	Subarachnoid hemorrhage	Admission ≥1 or outpatient department ≥2+ brain imaging (CT, MRI)
	I61	Intracerebral hemorrhage	
	I62	Other non-traumatic intracranial hemorrhage	
Gastrointestinal bleeding	K22.6	Gastro-esophageal laceration-hemorrhage syndrome	Admission ≥1 or outpatient department ≥2
			
	K25	Gastric ulcer	
	K26	Duodenal ulcer	
	K27	Peptic ulcer, site unspecified	
	K28	Gastrojejunal ulcer	
	K92.2	Gastrointestinal hemorrhage, unspecified	

*ICD, International Classification of Disease 10th Revision (ICD-10) code; CT, computed tomography; MRI, magnetic resonance imaging*.

### Cost-Effectiveness Analysis

A Markov chain decision-analysis model was constructed to evaluate the cost-effectiveness of DOACs compared with warfarin in intermediate stroke risk patients. We categorized health status into healthy with AF, post-health event (post-HF, post-IS, post-ICH, post-GI bleeding, or post-MI), and death. All health events, except for GI bleeding, were assumed to remain in the post-disease state or to transition to the death state. GI bleeding was assumed to transition to the healthy with AF state or death state (Supplementary Figure 2).

This study assumed the transition of health status in a 1-year cycle. Transient probabilities were determined by the annual incidence rates of five diseases (HF, MI, IS, ICH, and GI bleeding). The Markov chain decision-analysis model was repeated for 20 cycles. The discount rate was set as 4.5% (19).

This study took the perspective of a Korea healthcare system. All cost incurred from the point of view of Korean payers (medicine cost, cost due to health event) were included the cost consisted of medication cost, health event-related cost, and post-health event cost. Annual costs of medication were defined as 365 times the daily medication price. Health event-related cost was defined as the average admission cost for each health event. Post-event cost was estimated by subtracting the medication cost and event-related cost from the total costs. The willingness-to-pay (WTP) was set at $32,000 per quality-adjusted life-year (QALY) to reflect the South Korean gross domestic product ($31,494 in 2020). All costs were converted to USD (1 USD=1,000 KRW). We used the QALYs provided by previous studies (20–25).

### Sensitivity Analysis

Deterministic sensitivity analysis (DSA) was performed to examine which input parameter among those used in the above cost-effectiveness analysis most affects the model. In addition, probabilistic sensitivity analysis (PSA) was performed using a Monte Carlo simulation (10,000 times). The parameter used in sensitivity analysis shown in Supplementary Table 2.

All analyses were performed in TreeAge Pro 2020 (TreeAge Software, Inc., Williamstown, MA, USA) and SAS 9.4 (SAS Institute Inc., Cary, NC, USA).

## Results

The input parameter used to this analysis is shown in Supplementary Table 1.

We derived the annual incidence rate from a nationwide database. The annual incidence rate of IS was 0.75, 1.07, 1.50, 4.55, and 1.33% for warfarin, rivaroxaban, apixaban, dabigatran and edoxaban, respectively, with a CHA_2_DS_2_-VAS_c_ score = 1. Patients with CHA_2_DS_2_-VAS_c_ score = 2 had annual incidence rates of IS of 1.52, 0.64, 0.62, 0.53 and 0.64% for warfarin, rivaroxaban, apixaban, dabigatran and edoxaban, respectively. The utilities of warfarin, rivaroxaban, apixaban, dabigatran and edoxaban were 0.987, 0.994, 0.998, 0.970 and 0.998, respectively (Table 3). Their annual medication costs were $19.490, $795.420, $470.968, $870.890 and $670.010, respectively (Table 4).

**Table 3:** Fatality and utility used in base-case analysis.

**Model parameter**	**Base-case value (raw)**	**Base-case value (%/year)**	**Distribution**
**Fatality**			
Heart failure	4/1,223	0.32%	Beta
Myocardial infarction	0/61	0.00%	Beta
Ischemic stroke	2/92	2.17%	Beta
Intracranial hemorrhage	1/8	12.50%	Beta
GI bleeding	1/54	1.85%	Beta
**Utility**			
Warfarin (20)	0.987	0.987	Gamma
Rivaroxaban (20)	0.994	0.994	Gamma
Dabigatran (21)	0.970	0.970	Gamma
Apixaban (21)	0.998	0.998	Gamma
Edoxaban (21)	0.998	0.998	Gamma
Heart failure (22)	0.69	0.69	Beta
Myocardial infarction (23)	0.84	0.84	Beta
Ischemic stroke (24)	0.41	0.41	Beta
Intracranial hemorrhage (24)	0.56	0.56	Beta
GI bleeding (25)	0.70	0.70	Beta

*GI, gastrointestinal*.

**Table 4:** Cost used in base-case analysis.

**Model parameter**	**Base-case value (raw)**	**Base-case value (%/year)**	**Distribution**
**Cost($)**			
Medication cost (yearly)			
Warfarin	19.490	19.490	Gamma
Rivaroxaban	795.426	795.426	Gamma
Apixaban	470.968	470.968	Gamma
Dabigatran	870.890	870.890	Gamma
Edoxaban	670.010	670.010	Gamma
Event-related cost (per event)			
Heart failure	2,964.92	2,964.92	Gamma
Myocardial infarction	7,482.15	7,482.15	Gamma
Ischemic stroke	4,557.36	4,557.36	Gamma
Intracranial hemorrhage	7,108.77	7,108.77	Gamma
GI bleeding	1,583.03	1,583.03	Gamma

*GI, gastrointestinal*.

In the base-case analysis, the total cost of warfarin, rivaroxaban, apixaban, dabigatran and edoxaban in non-valvular AF patients was $2,874, $6,379, $5,151, $5,761 and $5,851, respectively (Table 5). The QALYs gained were 10.83, 10.95, 11.10, 10.49 and 10.99 years, respectively. The incremental cost-effectiveness ratios (ICERs) of rivaroxaban, apixaban, dabigatran and edoxaban (compared with warfarin) were $29,743.99, $8,426.71, –$8,483.04 and $18,483.55 each, respectively, and these estimates did not exceed the WTP threshold ($32,000 per QALY). Rivaroxaban, apixaban, and edoxaban were thus cost-effective compared with warfarin.

**Table 5:** Base-case analysis result comparing warfarin and DOACs.

**Strategy**	**Cost ($)**	**Incremental cost**	**QALY**	**ICER ($/QALY)**
Warfarin	2,874	Ref.	10.83	Ref.
Rivaroxaban	6,379	3,505	10.95	29,743.99
Apixaban	5,151	2,276	11.10	8,426.71
Dabigatran	5,761	2,886	10.49	−8,483.04
Edoxaban	5,851	2,976	10.99	18,483.55

*ICER, incremental cost-effectiveness ratio; QALY, quality adjusted life year; DOACs, direct oral antagonist oral anticoagulants*.

**Figure 1:**
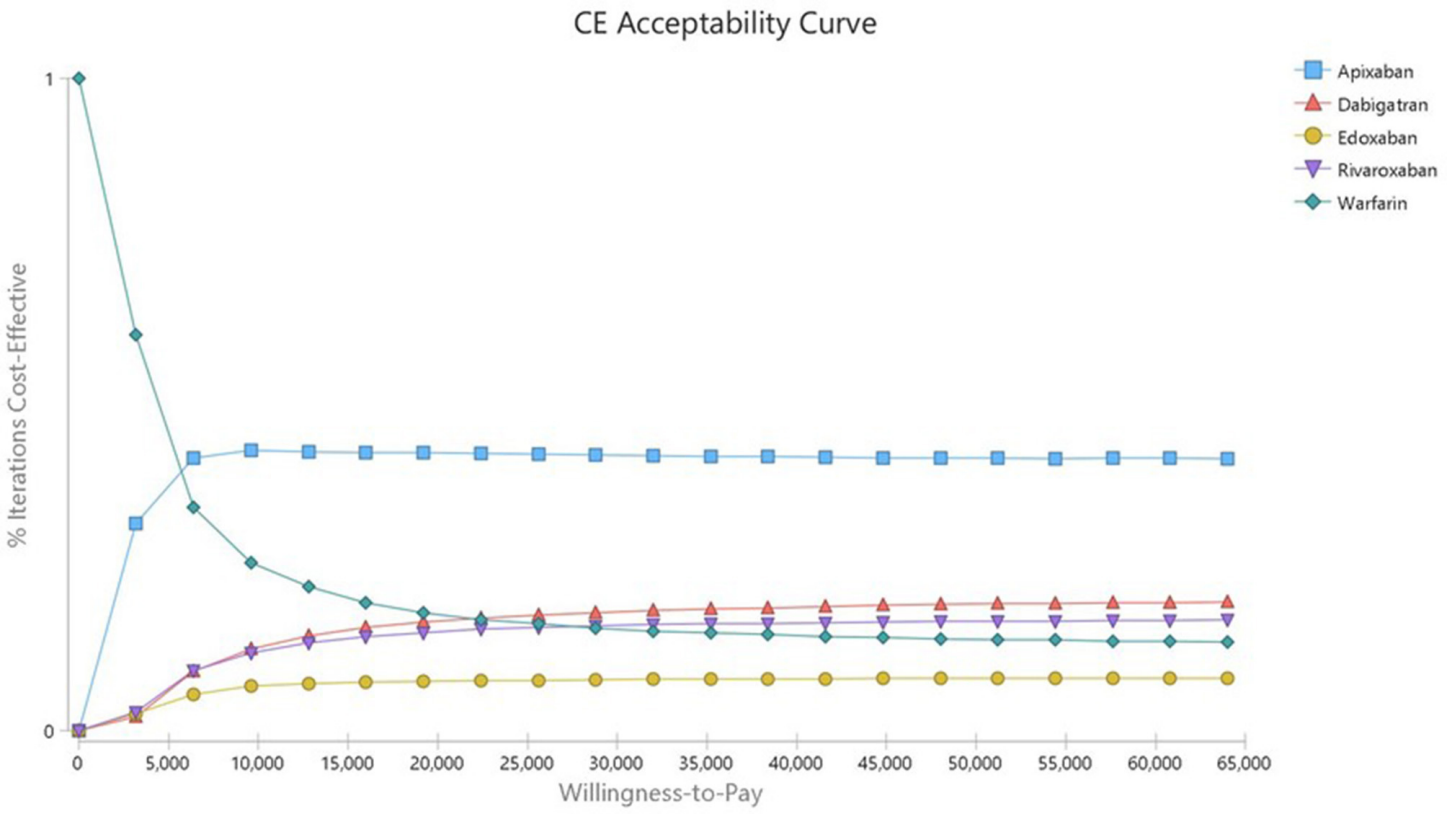
Cost-effectiveness acceptability curve of Warfarin vs. DOACs in AF patients with intermediate stroke risk.

In the deterministic sensitivity analysis, the input parameter most affecting the ICER was the drug cost in all analyses of DOACs compared with warfarin (Supplementary Figures 3–6). In a probabilistic sensitivity analysis, rivaroxaban, apixaban, dabigatran and edoxaban were cost-effective at 21.19, 49.73, 22.3, and 9.97% compared with warfarin, respectively (Table 6). In the acceptability curves for warfarin and DOACs, the ICERs of apixaban and rivaroxaban were lower than that of warfarin at the WTP ($32,000; Figure 1). The cost-effectiveness scatter plot is presented in Supplementary Figure 7.

**Table 6:** The PSA result of DOACs and warfarin.

	**Cost ($)**					**QALYs**				
	**Warfarin**	**Rivaroxaban**	**Dabigatran**	**Apixaban**	**Edoxaban**	**Warfarin**	**Rivaroxaban**	**Dabigatran**	**Apixaban**	**Edoxaban**
Mean ± SD	3,024 ± 218	6,431 ± 426	6,765 ± 495	5,132 ± 327	5,787 ± 383	10.72 ± 0.71	10.84 ± 0.71	10.87 ± 0.73	11.11 ±0.74	10.60 ± 0.69
Median	3,015	6,420	6,749	5,773	5,773	10.73	10.85	10.89	11.11	10.61
Minimum	2,333	4,967	5,152	4,463	4,463	7.76	7.88	8.31	8.31	7.83
Maximum	3,890	8,176	8,925	7,557	7,557	13.5	13.32	14.01	14.72	13.04
Sum	30,242,571	64,310,937	67,652,251	57,873,216	57,873,216	107,216.51	108,407.77	108,713.93	111,061.42	106,007.53
Size (n)	10,000	10,000	10,000	10,000	10,000	10,000	10,000	10,000	10,000	10,000
Variance	47,351	181,617	245,238	146,479	146,479	0.51	0.5	0.53	0.55	0.47
Variance/Size	5	18	25	15	15	0	0	0	0	0
SQRT (variance/size)	2	4	5	4	4	0.01	0.01	0.01	0.01	0.01

*SD, standard deviation; SQRT, square root; PSA, probabilistic sensitivity analysis; DOACs, direct oral antagonist oral anticoagulants; QALY, quality-adjusted life year*.

## Discussion

This study is the first to investigate the cost-effectiveness of DOACs compared with warfarin in South Korean non-valvular AF patients with intermediate stroke risk with fully independent from pharmaceutical company. In the base-case analysis, apixaban was the best alternative treatment to warfarin (with an ICER of $8,426.71). Rivaroxaban was also cost-effective compared with warfarin (with an ICER of $29,743.99). However, Dabigatran was not cost effective than warfarin (With an ICER of -$8,483.04). Although edoxaban exhibited an ICER of $18,483.55, it was not cost-effective compared with warfarin in the sensitivity analysis.

It is well known based on previous studies that the cost-effectiveness of DOACs is higher than that of warfarin (26, 27), which is consistent with our findings. Furthermore, the present study showed that apixaban and rivaroxaban were cost-effective compared with warfarin in both the base-case and sensitivity analyses (28, 29), which is consistent with previous studies (28–33). By contrast, our sensitivity analysis showed dabigatran and edoxaban to be less cost-effective than warfarin among patients with AF, which is not consistent with previous studies (33, 34). This discrepancy may stem from the difference in study population: the previous studies did not consider stroke risk (26, 27), while the present study focused on the intermediate stroke risk group.

There is little evidence in previous literature on the cost-effectiveness of DOACs in AF patients with intermediate stroke risk, and only a few investigated the cost-effectiveness of DOACs compared with warfarin while considering stroke risk (9, 17, 18). A UK study, which included 1,000 AF patients, showed that apixaban was more cost-effective than aspirin among AF patients with intermediate stroke risk, both having an ICER of $26,852 for a CHADS score of 1 and $14,001 for a CHA_2_DS_2_-VASc score of 1, respectively (17). However, our study included 7,954 AF patients with intermediate stroke risk, and the ICER of apixaban was $8,426.71 per QALY. A recent study from the Republic of Korea on AF patients with a CHA_2_DS_2_-VASc score of 1 (N=805) found that rivaroxaban was more cost-effective than warfarin (with an ICER of $98,051 per QALY) (9). The present study (N=7,954) included AF patients with intermediate stroke risk (having a CHA_2_DS_2_-VASc score of 1 in men 2 in women) and showed that the ICERs of apixaban and rivaroxaban were $8,426.71 and $29,743.99 per QALY, respectively.

In accordance with the previous studies, our findings clearly showed that DOACs were more cost-effective than warfarin. The higher cost-effectiveness of DOACs (vs. warfarin) in AF patients with intermediate stroke risk aligns with the current recommendations from Europe and the USA (13, 14). Nonetheless, DOACs are not subsidized in AF patients with intermediate stroke risk in many countries including the UK, USA, and China. In Korea, National Health Insurance program covers almost 100% of the Korean population (15). Thus, present study may provide evidence on the need for including AF patients with intermediate stroke risk as the beneficiary group of the DOACs' subsidy scheme.

Although our study included all AF patients in Korea during the study period and used real-world hospital utilization data to estimate cost-effectiveness, there are some limitations to be noted. First, the generalizability of the findings to other countries is limited since this study only included Koreans. Although many studies have been conducted in the West, they reported results similar to this study. Second, as an inherent issue of cost-effectiveness research, our findings are based on several assumptions. We assumed that the cost only included medication and health-related costs. Another assumption was that the transition probability evaluated the incidence of health events within 1 year. The other assumption was that we didn't consider health losses related to aging. To examine uncertainty, we performed a sensitivity analysis using Monte Carlo simulation. Third, in this study, the patients who had switched to the counter group had been excluded. The cost-effectiveness of warfarin depends on the time in therapeutic range (TTR), which was not evaluated in the present study (35). Warfarin users with poor TTR maintenance or minor bleeding episodes which were not included in clinical outcome, might underestimate cost of warfarin group. Fourth, because health events usually proceeded by systemic embolism and limitations of diagnosis existed in claims data. We could not consider systemic embolism as a final health event. Thus, considering the preventative effect of DOACs on systemic embolism (36, 37), our estimates might have been underestimated the cost-effectiveness of DOACs.

Lastly, only direct costs related to clinical events were considered in this study. As warfarin has a narrow therapeutic window and interactions with food or drugs, there may be indirect costs related to monitoring of the international normalized ratio, clinic visits, time spent during traveling and waiting, and consultation. This issue may have led to the underestimation of the cost-effectiveness of DOACs vs. warfarin.

In summary, we evaluated the cost-effectiveness of DOACs and warfarin using real-world hospital utilization data. The data of the study might be affected by reimbursement criteria of DOAC, not included the intermediate stroke risk patients in Korea. However, in this group of patients, the incidence of clinical events was substantial and DOAC has shown to be cost effective.

However, because it has been only a few years since DOACs were subsidized in South Korea, the long-term follow-up data are not sufficient to evaluate cost-effectiveness of DOACs and warfarin. Considering the result, the reimbursement scope of DOAC should be extended to the intermediate stroke risk patients who would benefit from DOAC. Therefore, future research on this topic might be necessary.

## Data Availability Statement

The raw data supporting the conclusions of this article will be made available by the authors, without undue reservation.

## Author Contributions

The health economic model was designed by J-BK and CK and performed by JChoi and WK. JChoi reviewed literature and drafted the manuscript, which was reviewed and revised by CK, J-BK, SS, and JCho. YK supported statistical method. Results were reviewed and interpreted by CK and J-BK. All authors contributed directly or indirectly to this study and agreed to manuscript submission.

## Funding

This study was supported by a Research Grant from the Korean Healthcare Technology R&D Project funded by the Ministry of Health and Welfare (HC19C0130) and Grant (2020-ER6301-00) from the Korea Disease Control and Prevention Agency in the Republic of Korea.

## Conflict of Interest

The authors declare that the research was conducted in the absence of any commercial or financial relationships that could be construed as a potential conflict of interest.

## Publisher's Note

All claims expressed in this article are solely those of the authors and do not necessarily represent those of their affiliated organizations, or those of the publisher, the editors and the reviewers. Any product that may be evaluated in this article, or claim that may be made by its manufacturer, is not guaranteed or endorsed by the publisher.
