# Supplementary material for: Cost-effectiveness of Direct Oral Anticoagulant vs. Warfarin Among Atrial Fibrillation Patients With Intermediate Stroke Risk
Source: Front Cardiovasc Med. 2022 Apr 11;9:849474. doi: 10.3389/fcvm.2022.849474 (PMC9035745; doi:10.3389/fcvm.2022.849474)
Supplement: Supplementary file 1 [file Data_Sheet_1.docx]

**Supplementary Data**

Supplementary Table 1 . Input parameter used in base-case and sensitivity analysis

| **Model parameter** | | | |  | | | **base-case value**  **(raw)** | | **base-case value**  **(%/year)** | **Distribution** |
| --- | --- | --- | --- | --- | --- | --- | --- | --- | --- | --- |
| Underlying condition(CVS) | | | | | |  |  | |  |  |
|  | 1 |  | | | | | 5087/7954 | | 63.95% | Dirichlet (Alpha) |
|  | 2 |  | | | | | 2867/7954 | | 36.05% | Dirichlet (Alpha) |
| proportion of the event | | | | | |  |  | |  |  |
|  | treatment effectiveness(%/year) | | | | | | |  |  |  |
|  | Heart Failure |  | | | | |  | |  |  |
|  | Warfarin | CVS=1 | | | | | 213/1330 | | 16.02% | Normal |
|  |  | CVS=2 | | | | | 79/461 | | 17.14% | Normal |
|  | Rivaroxaban | CVS=1 | | | | | 188/1123 | | 16.74% | Normal |
|  |  | CVS=2 | | | | | 95/629 | | 15.10% | Normal |
|  | Dabigatran | CVS=1 | | | | | 68/352 | | 19.32% | Normal |
|  |  | CVS=2 | | | | | 20/189 | | 10.58% | Normal |
|  | Apixaban | CVS=1 | | | | | 139/931 | | 14.93% | Normal |
|  |  | CVS=2 | | | | | 68/645 | | 10.54% | Normal |
|  | Edoxaban | CVS=1 | | | | | 223/1351 | | 16.51% | Normal |
|  |  | CVS=2 | | | | | 130/943 | | 13.79% | Normal |
|  | Myocardial infarction | |  | | | |  | |  |  |
|  | Warfarin | CVS=1 | | | | | 8/1330 | | 0.60% | Normal |
|  |  | CVS=2 | | | | | 6/461 | | 1.30% | Normal |
|  | Rivaroxaban | CVS=1 | | | | | 14/1123 | | 1.25% | Normal |
|  |  | CVS=2 | | | | | 4/629 | | 0.64% | Normal |
|  | Dabigatran | CVS=1 | | | | | 4/352 | | 1.14% | Normal |
|  |  | CVS=2 | | | | | 0/189 | | 0.00% | Normal |
|  | Apixaban | CVS=1 | | | | | 16/931 | | 1.72% | Normal |
|  |  | CVS=2 | | | | | 1/645 | | 0.16% | Normal |
|  | Edoxaban | CVS=1 | | | | | 17/1351 | | 1.26% | Normal |
|  |  | CVS=2 | | | | | 2/943 | | 0.21% | Normal |
|  | Ischemic stroke |  | | | | |  | |  |  |
|  | Warfarin | CVS=1 | | | | | 10/1330 | | 0.75% | Normal |
|  |  | CVS=2 | | | | | 7/461 | | 1.52% | Normal |
|  | Rivaroxaban | CVS=1 | | | | | 12/1123 | | 1.07% | Normal |
|  |  | CVS=2 | | | | | 4/629 | | 0.64% | Normal |
|  | Dabigatran | CVS=1 | | | | | 16/352 | | 4.55% | Normal |
|  |  | CVS=2 | | | | | 1/189 | | 0.53% | Normal |
|  | Apixaban | CVS=1 | | | | | 14/931 | | 1.50% | Normal |
|  |  | CVS=2 | | | | | 4/645 | | 0.62% | Normal |
|  | Edoxaban | CVS=1 | | | | | 18/1351 | | 1.33% | Normal |
|  |  | CVS=2 | | | | | 6/943 | | 0.64% | Normal |
|  | Intracranial hemorrhage | | | |  | |  | |  |  |
|  | Warfarin | CVS=1 | | | | | 2/1330 | | 0.15% | Normal |
|  |  | CVS=2 | | | | | 2/461 | | 0.43% | Normal |
|  | Rivaroxaban | CVS=1 | | | | | 2/1123 | | 0.18% | Normal |
|  |  | CVS=2 | | | | | 1/629 | | 0.16% | Normal |
|  | Dabigatran | CVS=1 | | | | | 1/352 | | 0.28% | Normal |
|  |  | CVS=2 | | | | | 0/189 | | 0.00% | Normal |
|  | Apixaban | CVS=1 | | | | | 0/931 | | 0.00% | Normal |
|  |  | CVS=2 | | | | | 0/645 | | 0.00% | Normal |
|  | Edoxaban | CVS=1 | | | | | 0/1351 | | 0.00% | Normal |
|  |  | CVS=2 | | | | | 0/943 | | 0.00% | Normal |
|  | GI Bleeding |  | | | | |  | |  |  |
|  | Warfarin | CVS=1 | | | | | 11/1330 | | 0.83% | Normal |
|  |  | CVS=2 | | | | | 3/461 | | 0.65% | Normal |
|  | Rivaroxaban | CVS=1 | | | | | 5/1123 | | 0.45% | Normal |
|  |  | CVS=2 | | | | | 3/629 | | 0.48% | Normal |
|  | Dabigatran | CVS=1 | | | | | 3/352 | | 0.85% | Normal |
|  |  | CVS=2 | | | | | 1/189 | | 0.53% | Normal |
|  | Apixaban | CVS=1 | | | | | 9/931 | | 0.97% | Normal |
|  |  | CVS=2 | | | | | 4/645 | | 0.62% | Normal |
|  | Edoxaban | CVS=1 | | | | | 8/1351 | | 0.59% | Normal |
|  |  | CVS=2 | | | | | 7/943 | | 0.74% | Normal |

*Abbreviations* CVS= CHA_2_DS_2_-VAS_c_ Score; GI= Gastrointestinal;

Supplementary Table 2. Input parameter used in sensitivity analysis

| **Model parameter** | |  | | | | **base-case value**  **(%/year)** | | **range** |  |
| --- | --- | --- | --- | --- | --- | --- | --- | --- | --- |
| Underlying condition(CVS) | | | |  | |  | |  |  |
|  | 1 | | | |  | 63.95% | | (0.6286-0.6501) |  |
|  | 2 | | | |  | 36.05% | | (0.3499-0.3711) |  |
| proportion of the event | | | |  | |  | |  |  |
|  | treatment effectiveness(%/year) | | | | | |  |  |  |
|  | Heart Failure | | | |  |  | |  |  |
|  | Warfarin | | | | CVS=1 | 16.02% | | (0.1521-0.1683) |  |
|  |  | | | | CVS=2 | 17.14% | | (0.1631-0.1797) |  |
|  | Rivaroxaban | | | | CVS=1 | 16.74% | | (0.1592-0.1756) |  |
|  |  | | | | CVS=2 | 15.10% | | (0.1431-0.1589) |  |
|  | Dabigatran | | | | CVS=1 | 19.32% | | (0.1845-0.2019) |  |
|  |  | | | | CVS=2 | 10.58% | | (0.099-0.1126) |  |
|  | Apixaban | | | | CVS=1 | 14.93% | | (0.1415-0.1571) |  |
|  |  | | | | CVS=2 | 10.54% | | (0.0987-0.1121) |  |
|  | Edoxaban | | | | CVS=1 | 16.51% | | (0.1569-0.1733) |  |
|  |  | | | | CVS=2 | 13.79% | | (0.1303-0.1455) |  |
|  | Myocardial infarction | | | |  |  | |  |  |
|  | Warfarin | | | | CVS=1 | 0.60% | | (0.0043-0.0077) |  |
|  |  | | | | CVS=2 | 1.30% | | (0.0105-0.0155) |  |
|  | Rivaroxaban | | | | CVS=1 | 1.25% | | (0.0101-0.0149) |  |
|  |  | | | | CVS=2 | 0.64% | | (0.0046-0.0082) |  |
|  | Dabigatran | | | | CVS=1 | 1.14% | | (0.0091-0.0137) |  |
|  |  | | | | CVS=2 | 0.00% | | (0-0) |  |
|  | Apixaban | | | | CVS=1 | 1.72% | | (0.0143-0.0201) |  |
|  |  | | | | CVS=2 | 0.16% | | (0.0007-0.0025) |  |
|  | Edoxaban | | | | CVS=1 | 1.26% | | (0.0101-0.0151) |  |
|  |  | | | | CVS=2 | 0.21% | | (0.0011-0.0031) |  |
|  | Ischemic stroke | | | |  |  | |  |  |
|  | Warfarin | | | | CVS=1 | 0.75% | | (0.0056-0.0094) |  |
|  |  | | | | CVS=2 | 1.52% | | (0.0125-0.0179) |  |
|  | Rivaroxaban | | | | CVS=1 | 1.07% | | (0.0084-0.013) |  |
|  |  | | | | CVS=2 | 0.64% | | (0.0046-0.0082) |  |
|  | Dabigatran | | | | CVS=1 | 4.55% | | (0.0409-0.0501) |  |
|  |  | | | | CVS=2 | 0.53% | | (0.0037-0.0069) |  |
|  | Apixaban | | | | CVS=1 | 1.50% | | (0.0123-0.0177) |  |
|  |  | | | | CVS=2 | 0.62% | | (0.0045-0.0079) |  |
|  | Edoxaban | | | | CVS=1 | 1.33% | | (0.0108-0.0158) |  |
|  |  | | | | CVS=2 | 0.64% | | (0.0046-0.0082) |  |
|  | Intracranial hemorrhage | |  | | |  | |  |  |
|  | Warfarin | | | | CVS=1 | 0.15% | | (0.0006-0.0024) |  |
|  |  | | | | CVS=2 | 0.43% | | (0.0029-0.0057) |  |
|  | Rivaroxaban | | | | CVS=1 | 0.18% | | (0.0009-0.0027) |  |
|  |  | | | | CVS=2 | 0.16% | | (0.0007-0.0025) |  |
|  | Dabigatran | | | | CVS=1 | 0.28% | | (0.0016-0.004) |  |
|  |  | | | | CVS=2 | 0.00% | | (0-0) |  |
|  | Apixaban | | | | CVS=1 | 0.00% | | (0-0) |  |
|  |  | | | | CVS=2 | 0.00% | | (0-0) |  |
|  | Edoxaban | | | | CVS=1 | 0.00% | | (0-0) |  |
|  |  | | | | CVS=2 | 0.00% | | (0-0) |  |
|  | GI Bleeding | | | |  |  | |  |  |
|  | Warfarin | | | | CVS=1 | 0.83% | | (0.0063-0.0103) |  |
|  |  | | | | CVS=2 | 0.65% | | (0.0047-0.0083) |  |
|  | Rivaroxaban | | | | CVS=1 | 0.45% | | (0.003-0.006) |  |
|  |  | | | | CVS=2 | 0.48% | | (0.0033-0.0063) |  |
|  | Dabigatran | | | | CVS=1 | 0.85% | | (0.0065-0.0105) |  |
|  |  | | | | CVS=2 | 0.53% | | (0.0037-0.0069) |  |
|  | Apixaban | | | | CVS=1 | 0.97% | | (0.0075-0.0119) |  |
|  |  | | | | CVS=2 | 0.62% | | (0.0045-0.0079) |  |
|  | Edoxaban | | | | CVS=1 | 0.59% | | (0.0042-0.0076) |  |
|  |  | | | | CVS=2 | 0.74% | | (0.0055-0.0093) |  |
| **Fatality** | | | | |  |  | |  |  |
|  | Heart Failure | | | |  | 0.32% | | (0.002-0.0044) |  |
|  | Myocardial infarction | | | |  | 0.00% | | (0-0) |  |
|  | Ischemic stroke | | | |  | 2.17% | | (0.0185-0.0249) |  |
|  | Intracranial hemorrhage | | | |  | 12.50% | | (0.1177-0.1323) |  |
|  | GI Bleeding | | | |  | 1.85% | | (0.0155-0.0215) |  |
| **Utility** | | | | |  |  | |  |  |
|  | Warfarin (20) | | | |  | 0.987 | | (0.953-0.995) |  |
|  | Rivaroxaban (21) | | | |  | 0.994 | | (0.990-0.997) |  |
|  | Dabigatran (21) | | | |  | 0.970 | | (0.960-0.980) |  |
|  | Apixaban (21) | | | |  | 0.998 | | (0.997-0.999) |  |
|  | Edoxaban (21) | | | |  | 0.998 | | (0.997-0.999) |  |
|  | Heart Failure (22) | | | |  | 0.69 | | (0.30-0.90) |  |
|  | Myocardial infarction (23) | | | |  | 0.84 | | (0.83-0.85) |  |
|  | Ischemic stroke (24) | | | |  | 0.41 | | (0.39-0.42) |  |
|  | Intracranial hemorrhage (24) | | | |  | 0.56 | | (0.54-0.58) |  |
|  | GI Bleeding (25) | | | |  | 0.70 | | (0.68-0.71) |  |
| **Cost($)** | | | | |  |  | |  |  |
|  | **Medication cost(yearly)** | | | |  |  | |  |  |
|  | Warfarin | | | |  | 19.490 | | (19.4-19.5) |  |
|  | Rivaroxaban | | | |  | 795.426 | | (794-797) |  |
|  | Apixaban | | | |  | 470.968 | | (470-472) |  |
|  | Dabigatran | | | |  | 870.890 | | (868-874) |  |
|  | Edoxaban | | | |  | 670.010 | | (669-671) |  |
| **Event-related cost (per event)** | | | | |  |  | |  |  |
|  | Heart Failure | | | |  | 2964.92 | | (2880-3050) |  |
|  | Myocardial infarction | | | |  | 7482.15 | | (7150-7810) |  |
|  | Ischemic stroke | | | |  | 4557.36 | | (4460-4650) |  |
|  | Intracranial hemorrhage | | | |  | 7108.77 | | (6270-6950) |  |
|  | GI Bleeding | | | |  | 1583.03 | | (1470-1700) |  |

*Abbreviations* CVS= CHA_2_DS_2_-VAS_c_ Score; GI= Gastrointestinal;


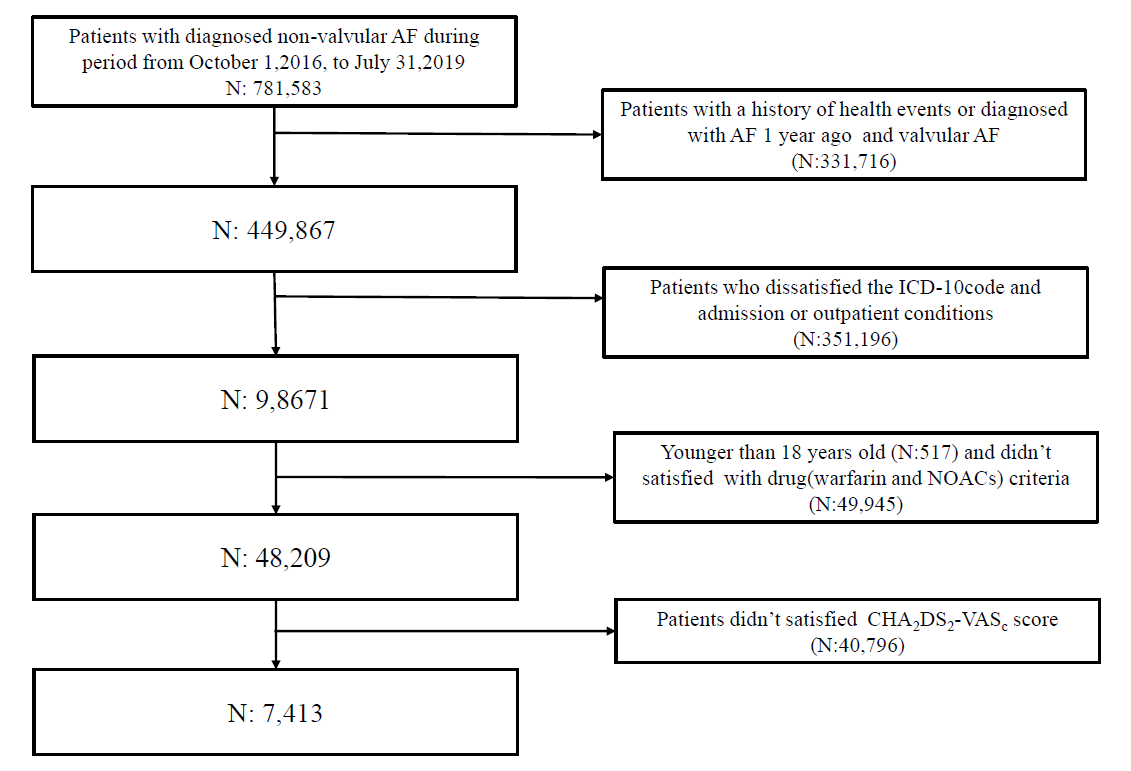


**Supplementary Figure 1. Flowchart of participants in AF patients with intermediate stroke risk**

*Abbreviations* AF = Atrial fibrillation; NOACs= Non-vitamin K antagonist oral anticoagulants; ICD = International Classification of Disease 10th Revision (ICD-10) codes


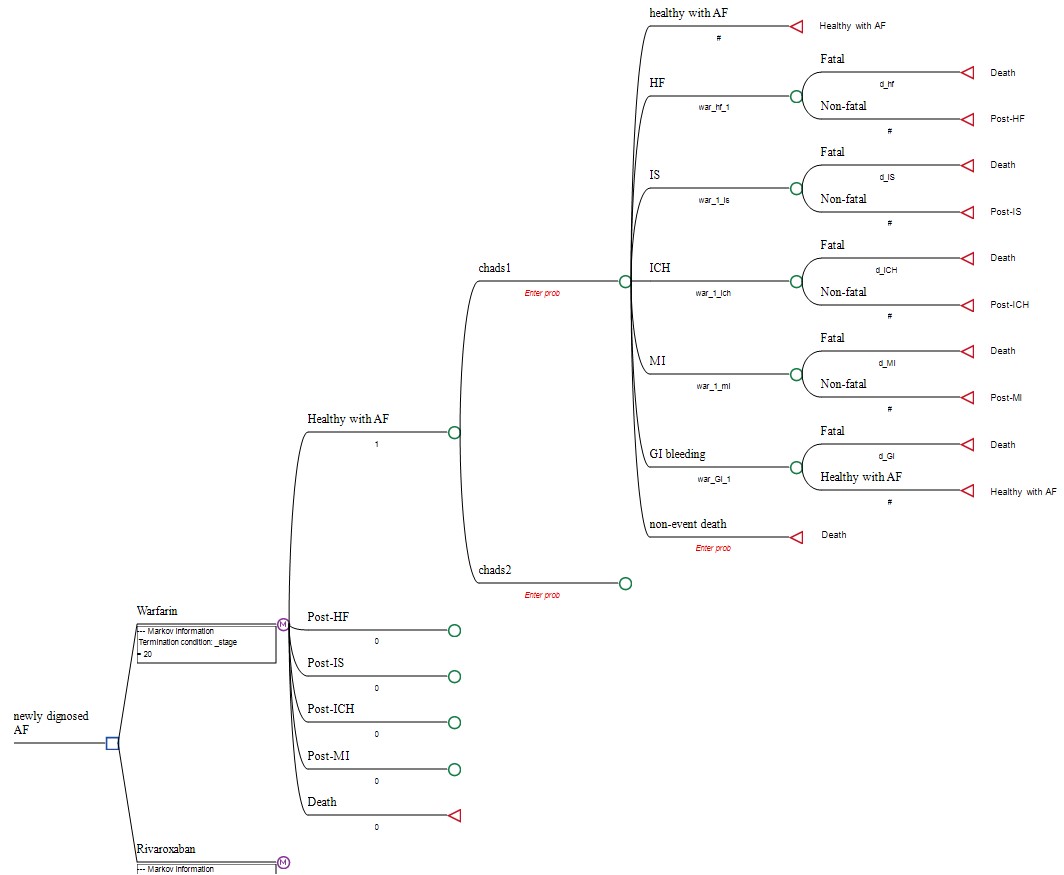


**Supplementary Figure 2. Decision tree model of cost effectiveness used in cost effectiveness analysis**

*Abbreviations* AF= Atrial Fibrillation; Chads = CHA2DS2-VASc score ; HF = heart failure ; IS = Ischemic stroke ; ICH = Intracranial hemorrhage ; MI = Myocardial infarction; GI = Gastrointestinal;


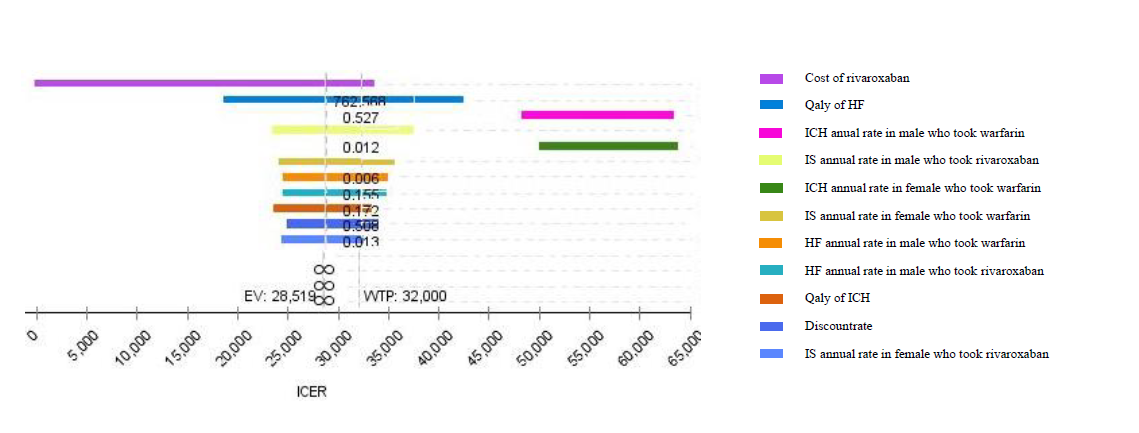
**Supplementary Figure 3. Result of Deterministic sensitivity analysis (Rivaroxaban versus warfarin)**

*Abbreviations* QALY = quality adjusted life year ; HF = heart failure ; IS = Ischemic stroke ; ICH = Intracranial hemorrhage ;


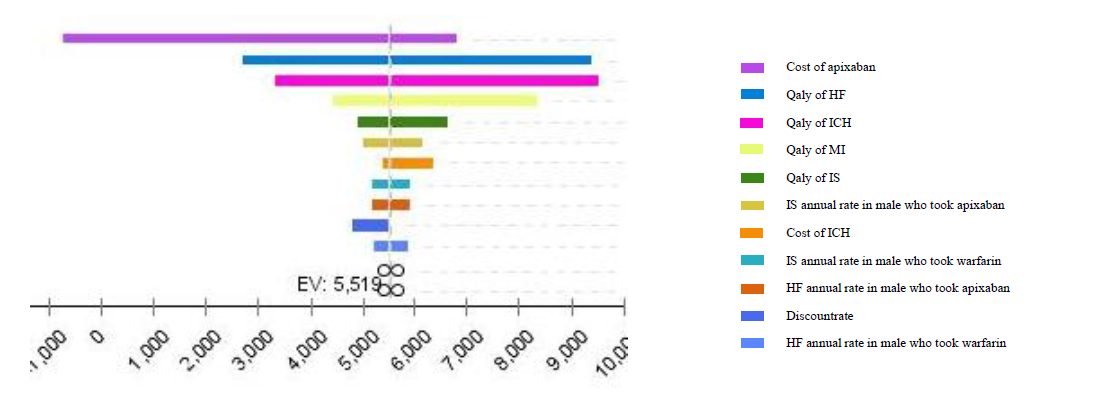
Supplementary Figure 4. Result of Deterministic sensitivity analysis (Apixaban versus warfarin)

*Abbreviations* QALY = Quality adjusted life year; HF = Heart failure; IS = Ischemic stroke; ICH = Intracranial hemorrhage; MI= Myocardial infarction;


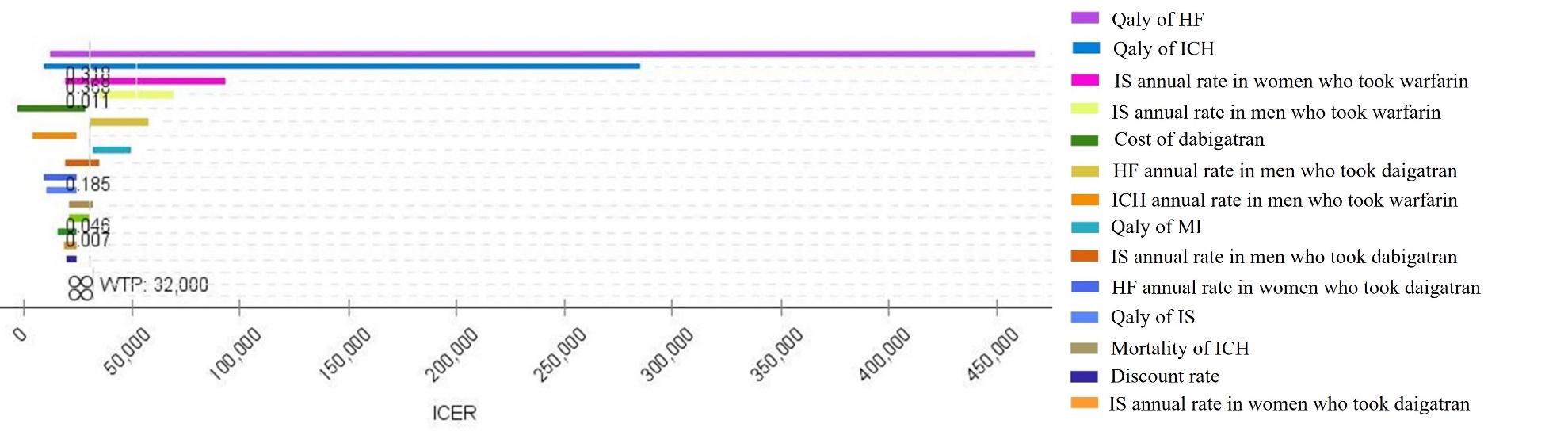


Supplementary Figure 5. Result of Deterministic sensitivity analysis (Dabigatran versus warfarin)

*Abbreviations* QALY = Quality adjusted life year; HF = Heart failure; IS = Ischemic stroke; ICH = Intracranial hemorrhage; MI= Myocardial infarction;


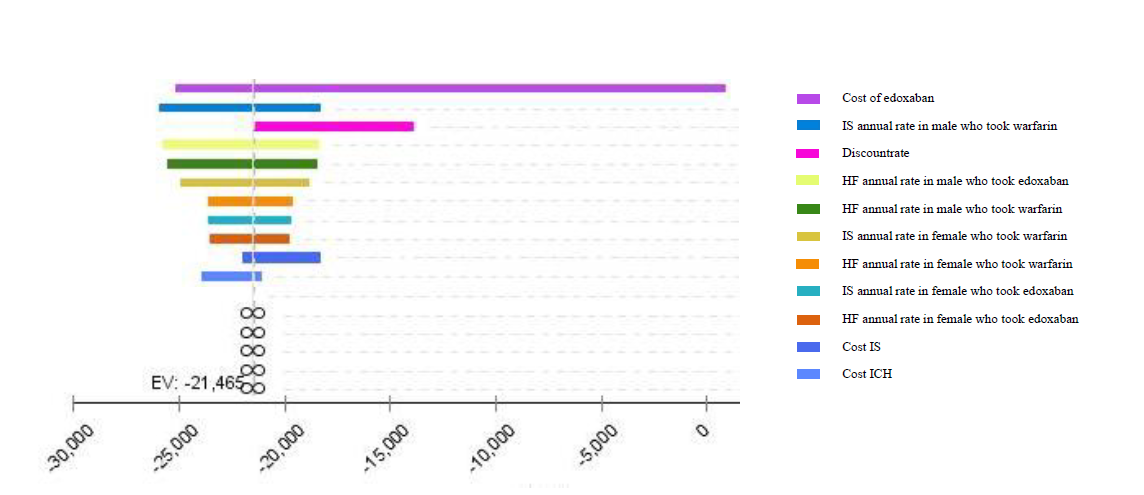
Supplementary Figure 6. Result of Deterministic sensitivity analysis (Edoxaban versus warfarin)

*Abbreviations* QALY = Quality adjusted life year; HF = Heart failure; IS = Ischemic stroke; ICH = Intracranial hemorrhage;


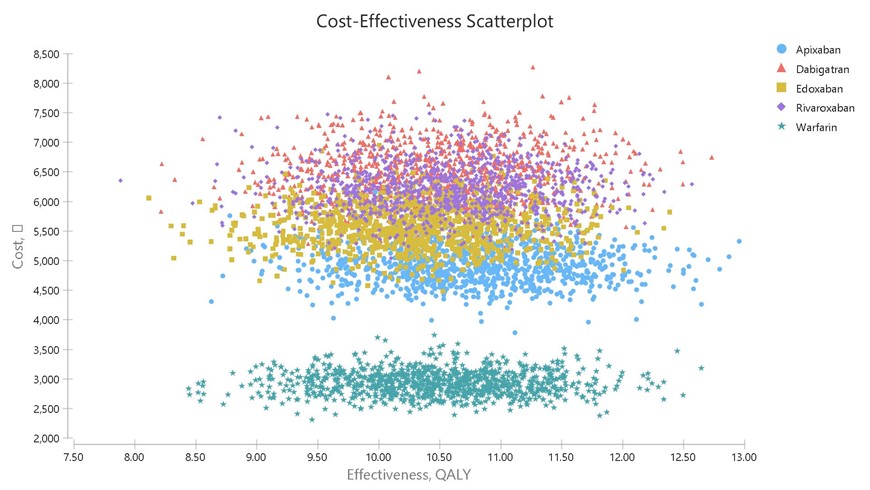


**Supplementary Figure 7. Cost-effectiveness scatterplot of Warfarin vs. DOACs in AF patients with intermediate stroke risk**

*Abbreviations* AF= Atrial Fibrillation; QALY = Quality adjusted life year;
